# Supplementary material for: Natural language processing and expert follow-up establishes tachycardia association with CDKL5 deficiency disorder
Source: Genet Med Open. 2023 Nov 18;2:100842. doi: 10.1016/j.gimo.2023.100842 (PMC11613813; doi:10.1016/j.gimo.2023.100842)
Supplement: Supplementary Material [file mmc1.pdf]

Natural language processing and expert follow-up establishes tachyarrhythmia association with *CDKL5* deficiency disorder

Supplementary files

Contents

Supplementary Figure 1. Cohort construction 2
Supplementary Figure 2. UMLS mapping process 3
Supplementary Figure 3. Human Phenotype Ontology term propagation 4
Supplementary Table 1. CDD and matched control cohort comparison........ 5
Supplementary Table 2. Clinical characteristics and cardiovascular phenotype of CDD cohort 6
Supplementary references 11

Supplementary Figure 1. Cohort construction

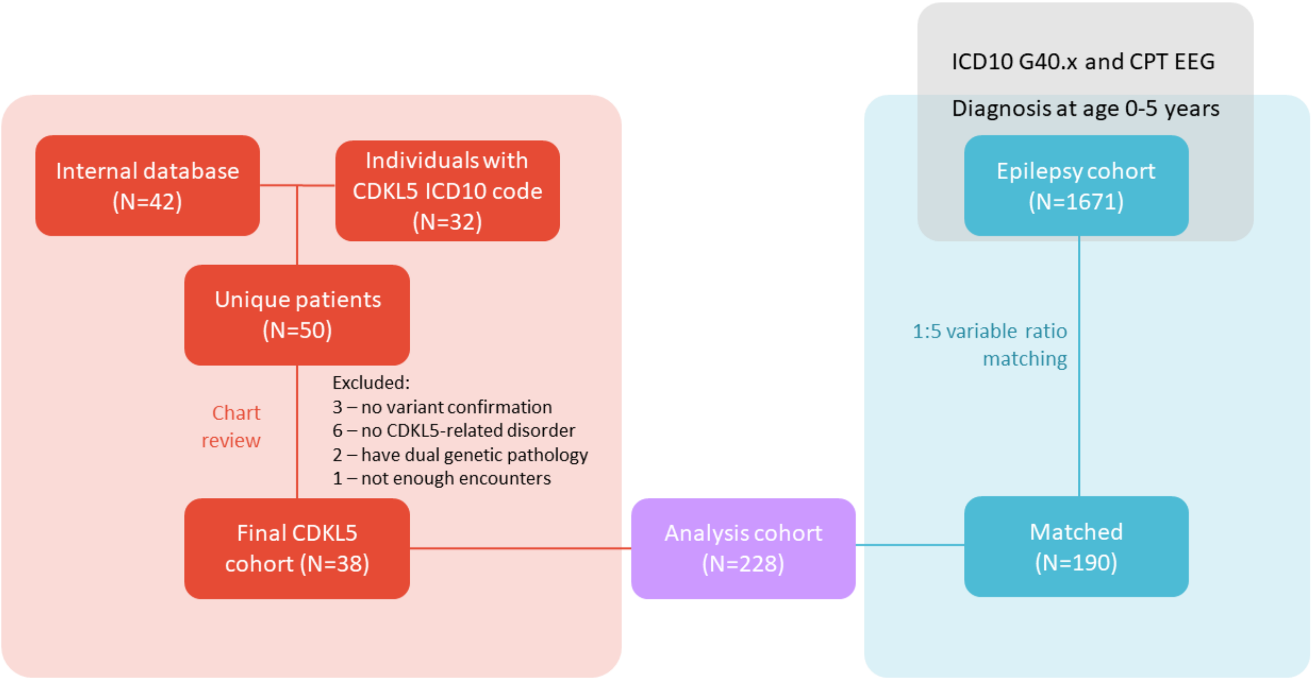

Figure S1. We included 42 people with CDKL5 deficiency disorder (CDD) listed in the internal database of the CDKL5 Center of Excellence at Cleveland Clinic Main Campus in Cleveland, Ohio. To ensure complete inclusion of individuals with CDD from other clinics of the Cleveland Clinic healthcare network, we additionally screened network-wide health records for the ICD-10 code G40.834 (Cyclin-Dependent Kinase-Like 5 Deficiency Disorder). The computational search identified eight additional patients. For the combined cohort of 50 people with CDD, we performed electronic chart review to verify the genetic diagnosis. Three individuals without genetic variant confirmation, six individuals who were coded with an ICD-10 code for CDD but turned out not to have the disorder, two having a concurrent pathogenic variant in another gene (*SCN1A*), and one patient for having only 1 encounter in the system were removed from the analysis. To construct the matched control cohort, we first identified 1671 individuals with epilepsy by querying the health record database for patient identifiers satisfying the following criteria: a) Having any ICD-10 G40 (“Epilepsy and recurrent seizures”) or ICD-9 345 code; b) Having a Current Procedural Terminology (CPT) code for any type of electroencephalography (EEG); c) Age 0-5 years at time of diagnosis (defined as an age of the first billing code for epilepsy). After nearest neighbor propensity score matching (see methods), we derived the matched control cohort of 190 individuals.

Supplementary Figure 2. UMLS mapping process

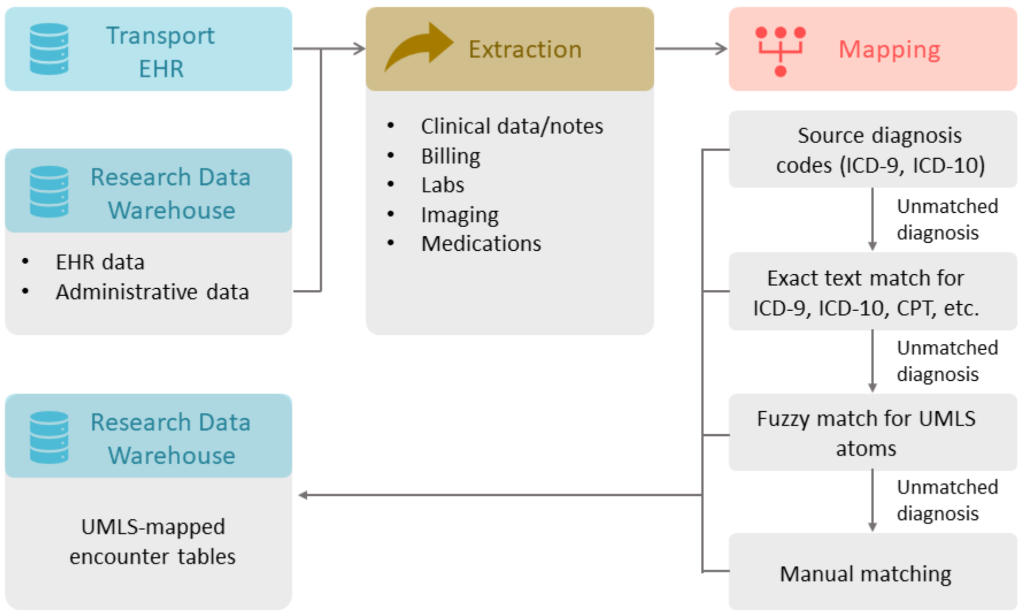

Figure S2. Research Data Warehouse (RDW) is an in-house relational database that hosts encounter tables mapped to Unified Medical Language System (UMLS, release 2022AA) concepts from different data sources. The data sources include electronic health record (HER; in this case, Epic) entries and administrative data sources hosted within RDW itself, as well as transport EHR (in this case, Golden Hour/emsCharts). Extracted data, which includes clinical notes, billing codes, laboratory and imaging results, and medication data is mapped to UMLS in several steps. At the first step, the source diagnosis codes, either ICD-9 or ICD-10, are directly mapped to UMLS. If the raw data entity does not include an associated diagnosis code, the next step involves mapping through exact unstructured text matching to same-source codes. Unstructured text, e.g. encounter notes, were analyzed with cTAKES[1], a natural language processing software, to extract medical phrases for mapping to UMLS and HPO. Remaining unmatched data entities undergo fuzzy matching for UMLS atoms, i.e., the smallest units of naming within UMLS system. All remaining unmatched data entities are manually mapped. Detailed description of database development is given by Reimer and Milinovich, 2020.[2]

Supplementary Figure 3. Human Phenotype Ontology term propagation

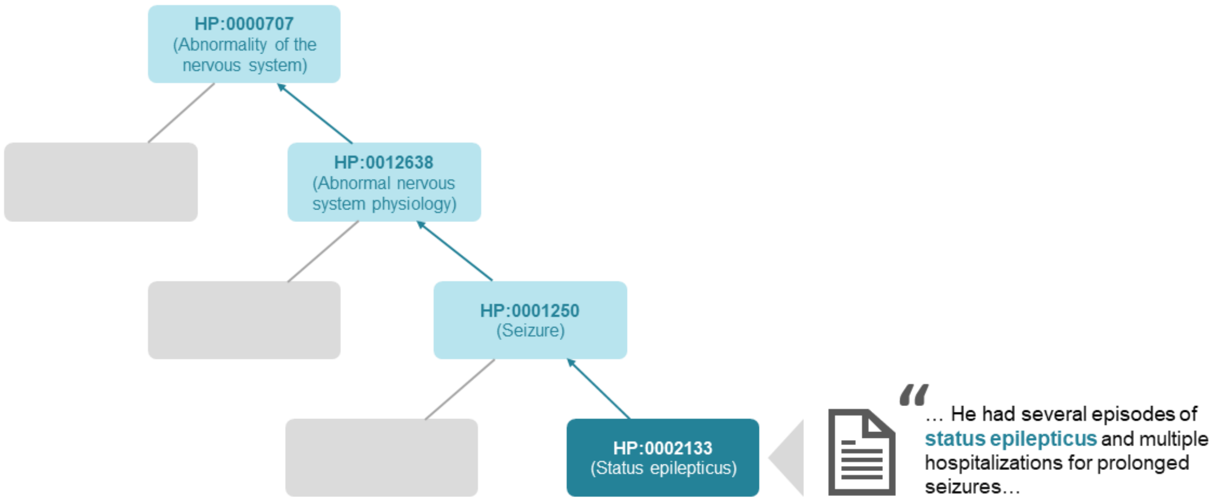

Figure S3. Human Phenotype Ontology (HPO) is a comprehensive hierarchical biomedical term library in which each term represents a more specific instance of a corresponding parent term. Propagation refers to identification of all parent terms of a particular term. Propagation facilitates phenotypic data harmonization and allows for phenotype exploration on different levels of specificity.

Supplementary Table 1. CDD and matched control cohort comparison

Variable		CDD	Control	P
Sex	Female, n(%)	28 (73.7)	132 (69.5)	0.746
	Male, n(%)	10 (26.3)	58 (30.5)	
Ethnicity	Hispanic or Latino, n(%)	3 (7.9)	14 (7.4)	1.000
	Not Hispanic or Latino, n(%)	35 (92.1)	176 (92.6)	
Number of encounters	Median (IQR)	38.0 (14.0 to 114.0)	22.0 (9.0 to 85.5)	0.357
Minimum encounter age	Median (IQR)	0.2 (0.0 to 1.1)	0.2 (0.0 to 1.3)	0.674
Median encounter age	Median (IQR)	3.4 (1.6 to 7.5)	4.3 (2.1 to 7.0)	0.630
Maximum encounter age	Median (IQR)	11.6 (6.5 to 16.3)	9.1 (4.9 to 19.1)	0.762
Length of follow-up	Median (IQR)	11.3 (5.0 to 16.0)	8.2 (2.6 to 19.1)	0.882

Supplementary Table 2. Clinical characteristics and cardiovascular phenotype of CDD cohort

ID	Sex	Variant	ACMG/AMP classification	Cardiac phenotype description	Autonomic features	Stimulant medications	Ambulatory status
1	Female	NM_003159.2:c.668T>G (NC_000023.11:g.18588067T>G) p.(Ile223Ser)	LP	Tachycardic, short PR interval	Drooling, irregular breathing	No	No
2	Female	NM_003159.2:c.529T>C (NC_000023.11:g.18584328T>C) p.(Tyr177His)	LP	Tachycardic	Drooling, urine retention, cold extremities	No	With support
3	Female	NM_001323289.2:c.2322del (NC_000023.11:g.18619912del) p.(Glu775Argfs*9)	P	Tachycardic	Drooling, cold extremities	No	No
4	Male	NC_000023.11(NM_003159.3):c.99+5G>A (NC_000023.11:g.18510859G>A; splice)	P	Hypertension and cardiomyopathy after treatment with ACTH for epileptic spasms. Deceased at teenage following cardiac arrest.	Drooling	No	No
5	Female	NM_003159.2:c.1498_1501dup (NC_000023.11:dup) p.(Leu501Glnfs*4)	LP	Tachycardic	Drooling	No	With support
6	Female	NM_001323289.2:c.2374dup (NC_000023.11:g.18619964dup) p.(Thr792Asnfs*9)	LP	Tachycardic	Drooling, cold	No	No

					extremities, urinary retention		
7	Female	NM_001323289.2:c.533G>T (NC_000023.11:g.18584332G>T) p.(Arg178Leu)	P	Tachycardic	None	No	No
8	Female	NC_000023.11:g.18582354_18626826del (deletion including exons 4-12)	P	Tachycardic	Drooling	No	Yes
9	Female	NM_001323289.2:c.533G>A (NC_000023.11:g.18584332G>A) p.(Arg178Gln)	P	ASD closed at teenage; Tachycardic	Drooling, cold feet	No	No
10	Male	NM_003159.2:c.351T>A (NC_000023.11:g.18579916T>A) p.(Tyr117*)	P	Neonatal SVT. Treated with propranolol until the 1.5 months of age. No SVT recurrence after discontinuation of propranolol. Hypertention due to treatment with ACTH. Tachycardic.	Drooling, cold feet	No	Yes
11	Female	NM_003159.2:c.623del (NC_000023.11:g.18588022del) p.(Gln208Argfs*20)	P	None	Drooling	Yes	Yes
12	Male	NM_003159.2:c.2323_2326del (NC_000023.11:g.18619913_18619916del) p.(Glu775Serfs*8)	P	C-section due to abnormal fetal rhythm (SVT) treated with beta-blockers	Drooling, cold extremities	No	No

				and flecainide; PFO			
13	Female	NM_003159.2:c.784T>C (NC_000023.11:g.18595387T>C) p.(Tyr262His)	LP	Tachycardic	Cold feet	No	No
14	Female	NM_001323289.2: c.364G>A (NC_000023.11:g.18579929G>A) p.(Ala122Thr)	P	Tachycardic, PFO	Drooling	No	No
15	Female	NM_001323289.2:c.1675C>T (NC_000023.11:g.18604599C>T) p.(Arg559*)	P	Tachycardic	Drooling, cold extremities	No	No
16	Male	NM_001323289.2:c.1976_1977del (NC_000023.11:g.18608842_18608843del) p.(Val659Glyfs*23)	P	Sinus bradycardia, followed by tachycardia	None	No	Yes
17	Male	NM_001323289.2:c.1165C>T (NC_000023.11:g.18604089C>T) p.(Gln389*)	P	Tachycardic and lQT syndrome. Died due to cardiorespiratory compromise.	Drooling, cold extremities, irregular breathing	No	No
18	Female	NC_000023.11(NM_003159.3):c.65-1G>A (NC_000023.11:g.18510819G>A; splice)	P	Tachycardic	Cold feet	No	Yes
19	Female	NM_001323289.2:c.1354C>T (NC_000023.11:g.18604278C>T) p.(Gln452*)	P	Tachycardic	Drooling, cold extremities	No	No
20	Female	NM_001323289.2:c.629del (NC_000023.11:g.18588028del) p.(Leu210Tyrfs*18)	P	Bradycardia, hypertension, LVH due to ACTH treatment for epileptic spasms; Tachycardic	None	No	With support
21	Female	NM_001323289.2:c.175C>T (NC_000023.11:g.18575383C>T) p.(Arg59*)	P	Tachycardic, PFO, right	None	Yes	No

				ventricular hypertrophy			
22	Female	NM_001323289.2:c.595T>C (NC_000023.11:g.18587994T>C) p.(Cys199Arg)	LP	Left ventricular hypertrophy	None	No	No
23	Male	NM_001323289.2:c.400C>T (NC_000023.11:g.18579965C>T) p.(Arg134*)	P	None	Irregular breathing, cold feet, drooling	No	Yes
24	Female	NC_000023.11(NM_003159.3):c.2046+1G>A (NC_000023.11:g.18608913G>A; splice)	LP	None	Drooling, cold feet	No	No
25	Male	NM_001323289.2:c.215T>C (NC_000023.11:g.18575423T>C) p.(Ile72Thr)	P	None	None	No	No
26	Male	NM_001323289.2:c.608A>G (NC_000023.11:g.18588007A>G) p.(Glu203Gly)	LP	None	Drooling	No	No
27	Female	NM_001323289.2:c.412C>G (NC_000023.11:g.18581899C>G) p.(Pro138Ala)	P	None	None	No	No
28	Female	NM_001323289.2:c.470C>T (NC_000023.11:g.18584269C>T) p.(Ala157Val)	LP	None	None	No	No
29	Male	NM_001323289.2:c.587C>G (NC_000023.11:g.18587986C>G) p.(Ser196Trp)	LP	None	Drooling	No	No
30	Female	NM_001323289.2:c.934A>T (NC_000023.11:g.18598570A>T) p.(Leu312*)	P	None	Irregular breathing	No	Not applicable (too young)
31	Female	NM_001323289.2:c.46del (NC_000023.11:g.18507142del) p.(Val18Leufs*2)	P	None	None	No	Not applicable (too young)
32	Female	NM_001323289.2:c.725del (NC_000023.11:g.18588124del) p.(Pro242Leufs*25)	P	None	None	No	No
33	Female	NC_000023.11:g.18209153_19223560del	P	None	None	No	No

34	Female	NC_000023.11(NM_001323289.2):c.65-2A>T (NC_000023.11:g.18510818A>T; splice)	P	None	Cold feet	No	No
35	Female	NM_001323289.2:c.1244_1245del (NC_000023.11:g.18604168_18604169del) p.(Thr415Argfs*3)	LP	None	Drooling	No	No
36	Female	NM_001323289.2:c.1988del (NC_000023.11:g.18608854del) p.(Ser563Thrfs*121)	LP	None	Drooling	No	No
37	Male	NM_001323289.2:c.103A>C (NC_000023.11:g.18564480A>C) p.(Thr35Pro)	LP	None	None	No	No
38	Female	NC_000023.11:g.18564468_18564474delinsGCAGA (splice)	P	None	Drooling	No	Yes

Abbreviations: ACTH, adrenocortocotropic hormone; ASD, atrial septal defect; ECG, electrocardiogram; LP, likely pathogenic; LVH, left ventricular hypertrophy; lQT, long QT syndrome; P, pathogenic; PFO, patent foramen ovale; SVT, supraventricular tachycardia.

Supplementary references

1. Savova GK et al. Mayo clinical Text Analysis and Knowledge Extraction System (cTAKES): architecture, component evaluation and applications. *J Am Med Inform Assoc.* 2010 Sep-Oct;17(5):507-13. doi: 10.1136/jamia.2009.001560.
2. Reimer AP, Milinovich A. Using UMLS for electronic health data standardization and database design. *J Am Med Inform Assoc*. 2020;27(10):1520-1528. doi:10.1093/jamia/ocaa176
